# Supplementary material for: Are carbon emissions trading and green financial instruments synergistic? -Comprehensive quantitative research based on content analysis
Source: PLoS One. 2024 Mar 7;19(3):e0298601. doi: 10.1371/journal.pone.0298601 (PMC10919601; doi:10.1371/journal.pone.0298601)
Supplement: S1 Data — (ZIP) [file pone.0298601.s001.zip › Data/地级市Z_F工作报告.zip!/╔╜╬≈/╜·│╟╩╨/└┤╫╘╣1⁄2╓┌║┼í╛┬φ┐╦╩2╛▌═°í┐_2017.docx]

各位代表：

　　现在，我代表市人民政府向大会报告工作，请予审议，并请市政协委员和其他列席人员提出意见。

　　一、2016年工作回顾

　　2016年，新一届政府组成以来，在省委、省政府和市委的坚强领导下，在市人大、市政协的监督支持下，市政府紧紧依靠全市人民，深入贯彻落实党的十八大和十八届三中、四中、五中、六中全会精神和习近平总书记系列重要讲话精神，按照省委“一个指引、两手硬”的重大思路要求和市委“一争三快两率先”战略部署，坚持“大开放、大转型、大创新”三大发展思路，以谋划推进“十大战略工程”为重点，践行发展新理念、适应发展新常态，统筹推进稳增长、促改革、调结构、惠民生、防风险各项工作，在极为艰难的形势下，实现了“下半年好于上半年，2016年好于上年”的努力目标。全年生产总值完成1049.3亿元，同比增长3.9%；规模以上工业增加值增长2.5%；固定资产投资完成1150.4亿元，增长4.1%；社会消费品零售总额完成386.2亿元，增长7.6%；一般公共预算收入完成89.3亿元，下降4.9%；外贸进出口总额完成6.0亿美元，下降32.8%；城镇居民人均可支配收入完成28223元，增长5.9%；农村居民人均可支配收入完成11635元，增长6.6%。

　　我们主要做了以下工作：

　　——发展思路更加清晰。明确提出了“十三五”时期确立的“三大”发展思路和大干快干的“十大战略工程”。紧紧围绕“十三五”时期建设“双百”规模大市区目标，明确提出了把环城高速作为外环，207国道、陵沁路改线作为市区二环，以现行的西环、北环、东207改线、南白水街作为内环的“三环”城市道路骨架新格局，确定了今年全力推进的“双十”城市建设工程。紧紧围绕转型综改、创新驱动经济工作的主线，制定了《2017·“1533”工业转型升级攻坚年行动方案》。启动了《美丽晋城战略规划》和《全域旅游发展五年规划》编制工作。

——精准施策收效明显。面对经济下行压力，持续实施年初制定的《2016·“1533”工业行动方案》，按照方案的九条政策措施，实行“三个一”运行监测服务。建立政银企常态化对接机制，开展干部入企服务活动，有力促进了企业稳定生产，实现了工业经济稳步回升。规模以上工业增加值累计增速从10月份开始由负转正。

——潜力活力显著增强。与省农发行签署200亿元的战略投资合作协议，与中船重工集团签署110亿元的项目投资协议。与省晋能集团、省能交投集团、省民航局、省金控集团进行项目对接。三次赴深圳与郭台铭总裁会谈，就富士康高技术玻璃项目落户晋城达成意向。组织企业家和市直有关部门赴上海自贸区、苏州工业园学习考察，引进上海临仕激光科技公司、上海迪威尼生物制造两家企业落户晋城。派6名同志赴上海商务部国家产业转移中心、上海漕河泾开发区、张江高科技开发区、松江开发区进行挂职招商。聘请郭台铭总裁、范守善院士和霍国庆教授担任市“双创”促进会顾问，聘请上海漕河泾开发区元老陈青洲博士担任晋城市政府产业发展总顾问，聘请南开大学旅游与服务学院院长白长虹为晋城市政府旅游顾问。

——新的动能正在蓄积。兰花20万吨己内酰胺、纳米碳酸钙项目正式投产，清慧5000万件轨道交通新型材料项目、山西迪威尼生物高科技制造项目、中船重工风电总装维护基地项目开工建设，富士康摩拜单车项目产品正式下线。晋城海关、晋城商检、兰花保税物流中心正式运营。积极与国家发改委、交通部、民航总局等对接民用机场前期建设事宜。陵沁路西北环改线工程正式通过省交通厅评审。

——民生改善持续提高。全年民生领域财政支出完成143亿元，占总支出的82.6%。旅游职业教育集团挂牌成立，省定12所新建改建农村幼儿园全部完工，沁水县通过了国家义务教育发展基本均衡督导评估认定。晋城大医院新门诊楼正式投入使用。在全省率先启用居民健康卡“一卡通”，最早启动失业保险“四项补贴”的申领发放。市区提前半个月实施集中供暖。新农合人均筹资标准由470元提高到540元，参合率达99.31%。新增3种医保门诊慢性病病种，病种数、待遇水平均在全省领先。城乡低保标准每人每月提高30元。农村危房改造达到2370户，新开工城市棚户区改造6045套。安全生产保持平稳态势。平安晋城建设深入推进，在全省公安机关人民群众安全感和满意度调查中位居第一，连获“五连冠”。国防动员和双拥工作扎实开展，第三次荣膺“全国双拥模范城市”称号。率先在全省提前三年完成“市志”、“年鉴”两全目标。同时，妇女儿童、民族宗教、外事侨务、档案、统计、气象、人防、地震、红十字、慈善、残疾人、老龄等各项事业都取得了新的成绩。

——干事创业劲头更大。换届以来，广大干部在“两学一做”学习教育中得到锤炼，干部的精气神有了新的提振。城区广大干部在春节大年三十连夜奋战，一举拆除影响景西北路全线通车一年半的原太印厂区。市工商局提高办事效率，一个半小时将营业执照送到招商引资的企业手中。市发改、经信、财政、规划、住建、交通、旅游等多个部门，春节期间加班加点定任务、赶进度，促进了今年各项工作的早部署、早安排。大家倍加勤奋工作的劲头和释放出的力度大、节奏快、效率高的能量，正在成为全市广大干部自觉的行动，大家争先竞位、勇争第一的工作标准，已经初步形成了广大干部自觉信守的思想共识。我们要十分珍惜这种正能量，要把这种正能量凝聚成一股强大的“文化磁场”，辐射人人，激增倍长，让每一个干部、每一份事业都连点成线，以线带面，朝向大业，快步而行，着眼正道，全力拼搏，决不辜负全市人民群众的信任和期望！

　　各位代表，换届以来，新一届政府同心同德、齐心协力，解放思想、开拓创新，以上率下、迎难而上，积极应对各种挑战，取得了一定的成绩。这些成绩的取得，是省委、省政府和市委正确领导的结果，是市人大、市政协有效监督、大力支持的结果，是全市人民信任、支持和齐心协力、共同奋斗的结果。在此，我代表市人民政府，向全市人民，向驻市解放军指战员、武警官兵和公安干警，向各民主党派、各人民团体和各界人士，向各位老领导、老同志，向所有关心、支持晋城发展的同志们、朋友们，致以崇高的敬意和衷心的感谢！

　　同时，我们也清醒地认识到，我市仍处于爬坡过坎、转型攻坚的关键时期，发展中还存在不少困难和问题:思想解放不够，创新意识不强；产业结构单一，新兴产业和高科技项目严重缺乏；财政收入结构不优，收支矛盾日益凸显；基础设施建设和民生事业仍是短板；环境保护、安全生产、金融风险等隐患仍然存在；发展环境不优、体制机制不活等问题还不同程度存在；一些部门的服务意识、落实能力、主动担当的劲头与发展要求和人民期盼还不适应。对此，我们一定要高度重视、认真加以解决。

　　二、2017年工作安排

　　2017年，是实施“十三五”规划的关键之年，是推进供给侧结构性改革的深化之年，也是落实市第七次党代会战略部署的开局之年。认真做好今年的政府工作，任务艰巨，责任重大，使命光荣。

　　今年政府工作的总体要求是：深入贯彻习近平总书记系列重要讲话精神和党中央治国理政新理念新思想新战略，按照省委“一个指引、两手硬”的重大思路和要求，以及市委“一争三快两率先”战略部署，坚持稳中求进总基调，坚持“大开放、大转型、大创新”发展思路，以“转型升级攻坚年”为主题，着力深化“供改”和“综改”，提高发展质量和效益，加快创新驱动、转型升级，促进经济稳步向好和社会和谐稳定，在决胜全面小康的新征程中迈出坚实步伐，以优异成绩迎接党的十九大胜利召开。

　　主要预期目标：地区生产总值同比增长5.5%左右，规模以上工业增加值增长3%，固定资产投资增长目标根据新的统计口径确定，公共财政预算收入增长1%，社会消费品零售总额增长7.5%，城镇居民人均可支配收入增长6%、农村居民人均可支配收入增长6.5%，居民消费价格涨幅控制在3%左右，城镇新增就业3.4万人，城镇登记失业率控制在4.2%以内。资源节约、环境质量、民生改善等约束性指标控制在省下达指标以内。

　　做好今年的政府工作，要重点围绕“转型升级攻坚年”主题，全力实施好“十大行动”。即产业转型升级、开发区改革创新、招商选资引智、城建质量提升、通道快捷建设、全域旅游打造、精准脱贫攻坚、高端人才引进、生态环境治理、民生福祉改善十大行动。实施“十大行动”是推进落实“十三五”时期“十大战略工程”在今年的具体化。完成好“十大行动”，必须坚持深化“供改”和“综改”的总要求，把“供改”和“综改”工作的主线全方位、全过程贯穿到“十大行动”中。完成好“十大行动”，必须要有落实的高标准、完成的硬指标，我们把检验“十大行动”执行的标准和目标定位为“五化三型三量齐升”。即，产业升级必须具体到项目化，开发区改革必须达到市场化，招商引资必须聚焦精准化，全域旅游必须实现标准化，城市提质必须推进规模化；“三型”的标准，就是产业体系要初显“创新型”、城市规模要初显“双百”型、生态环境要初显“园林型”，最终使全市的经济总量、人均均量、运行质量“三量”齐升。“五化三型三量齐升”是检验“十大行动”落实的硬杠杆、硬标准，也是完成“十大行动”任务的最高要求，更是向全市人民的庄严承诺。广大干部必须要在落实“十大行动”中出实招、见功夫、见智慧、见担当；必须全力扛起落实“十大行动”的重任，以不服输、敢胜利的“精气神”忠诚担当、攻坚克难，不折不扣把“十大行动”落实到底，向全市人民交出一份优秀的答卷！

　　（一）产业转型升级行动。

　　产业转型升级是资源型地区走出转型升级跨越发展新路的根本标志。今年，要以十大转型项目建设为主要抓手，把深化供给侧结构性改革作为加快产业转型升级的根本途径，以项目调结构、以项目促转型，以改革促升级、以创新增规模，扎实推进产业转型升级行动。全力推动十大转型项目开工建成投产。一是富士康光学相机模组设计线项目要加快完成设计生产线工程设备件的转移规划，年内完工投入运行；二是富士康纳米光机电研发中心要于9月份建成，研发中心工作组5月份进驻中心；三是富士康高技术玻璃项目要加快完成从意向到实地勘查的进程；四是中船重工风电总装基地要实现首台套设备下线；五是清慧5000万件轨道新型材料要于9月份建成投产；六是上海临仕激光再制造项目年底前建成激光焊接、切割、熔覆三条生产线；七是山西迪威尼生物高科技制造项目10月份要投产；八是国家量子协会大数据项目要完成一期工程；九是晋钢铸造产业园铸钢件工业升级10月份要试产；十是晋煤华昱煤电化循环工业园于11月份要试产。这十大项目是今年产业转型升级行动的标杆项目，是全市“转型升级攻坚年”的风向标。十大项目的选择，突出了战略性新兴产业的方向和高科技项目的要求。富士康相机模组设计线是相机模组领域世界级的设计工艺标准；富士康光机电研发中心是符合国家工信部“中国2025”制造中心准入的研发中心；富士康高技术玻璃项目是目前国际最高等级的玻璃材料制造；中船重工、清慧制造、临仕激光再制造项目均为新能源、高端装备制造、生物高科技制造和新材料类别高新产业；山西迪威尼生物高科技的四个项目均为国内空白，世界领先，为国家环保部重点实验室项目；大数据项目是适应5G时代发展的集云计算、互联网、物联网等一体的数字经济产业；晋钢铸造园是提升现有传统产品，为高附加值产品的转型升级项目；晋煤煤电化项目为利用晋城“三高”煤的废旧利用推进循环经济发展的产业项目。这十大项目是市政府全力狠抓的重大转型升级项目。十大项目的建设，必将对全市工业企业走出资源型地区转型升级跨越发展的新路起到巨大的引领作用。富士康、中船重工、晋煤、晋钢等十大企业要挂图作战、倒排工期，列出投资进度，抓工期、抓进度、抓质量，确保按期开工、按期建成、按期投产。市直各部门和各县（市、区）、开发区要树立服务是永恒的主题、创新是永恒的灵魂、发展是第一要务的理念，切实做好项目服务工作，确保项目有序快速推进。各县（市、区）也要结合各自实际确定转型项目，形成狠抓转型项目谋划、转型项目引进、转型项目建设的强大效应，形成你追我赶、时不我待的转型劲头，争当转型升级的排头兵。全市的企业和企业家要积极围绕市场研究项目，围绕产业谋划项目，围绕发展推进项目，不断增强企业的核心竞争力。要以十大转型项目开工建成投产，搭起晋城转型升级的骨架，迅速把项目变为晋城经济版图新的增长点和增长极。全力推动深化国企国资改革任务全面完成。紧紧围绕省委骆惠宁书记提出的六大国企国资改革的要求，严格按照“分级实施，归口负责，组建平台，一企一策”的基本思路，5月份要出台《晋城市深化国企国资改革的指导意见》，9月底前要出台晋城市国企国资改革的配套政策和实施办法，下半年全面完成国企国资改革任务。年内完成国有资本投资运营公司组建并投入运营，构建“国资监管机构—国有资本投资运营公司—国有企业”三个层面的国资监管新体系，大力推动兰花集团等优势骨干企业在全市国企国资改革中领先一步。全力推动传统产业提升改造。要引导传统产业提高技术水平、提高制造能力、优化产品结构、拉长产业链条。煤炭产业，要提高煤炭先进产能占比，抓好科兴赵庄、煤销盛泰、阳泰武甲、晋煤胡底、晋圣三沟鑫都、晋圣松峪、晋圣固隆、天安盈盛、天安壁盈、天安苇町、天安宏祥11座矿井联合试运转和竣工转产工作，新增产能1000万吨/年以上，新增先进产能30%以上。紧紧盯住清洁高效利用目标，重点把中煤集团国投里必400万吨煤矿建成清洁高效利用的先进产能综合示范矿井。冶铸产业，要按照《2017·“1533”工业转型升级攻坚行动方案》要求，抓好10家中小型铸造企业的技改工作，全力推动冶铸企业由粗放生产向循环经济发展，由铸造向制造发展，重点抓好晋钢铸造产业园项目建设。要立足现有铸造集聚区，引导铸造企业向园区集中，提高产业集聚水平。南村各铸造企业今年要全部实施煤改气和环保设施改造。要促进铸造产品提档升级，抓好泫氏实业年产40万吨大口径球墨铸管项目建设。电力产业，要积极调整电力结构，对容量小、煤耗高、排放不达标、单机20万千瓦以下的火电装机，要分类分年代逐步淘汰。对单机30万千瓦以上的火电装机，要实施超低排放节能改造。要进一步扩大电力直接交易规模，适当发展有比较优势的高载能产业。重点抓好中船重工30万千瓦风力发电，阳城大连尚能、恒瑞两个15万千瓦风力发电项目，泽州华电、沁水远景汇合、中电投陵川三个10万千瓦风力发电项目和金驹煤电化胡底瓦斯发电项目建设。煤化工产业，要以煤基新材料和煤基新能源为发展方向，全力攻关煤基领域科技创新链，加快谋划一批现代化高端新型煤化工产业项目，把进入产业的各种资源“吃干榨尽”。

　　各级各部门要认真落实市政府出台的《2017·“1533”工业转型升级攻坚年行动方案》。《方案》明确了培育、支持、奖励的方向，明确了从科技高度、建设进度、投资强度、园区建设、总产值增幅、技术中心、企业上市、技术改造等八方面服务举措。在经信委设立了服务企业办公室，实行“五个一”服务工作机制，即每月用十天时间到企业服务，每月清零一批企业反映的问题，每季度召开一次调度会议，每半年进行一次评估，全年进行一次奖励兑现。各县（市、区）、市直各部门都要积极为企业提供优质服务，用“三大”发展思路营造一流的投资发展环境，真正把晋城建设成投资的洼地、转型的高地。同时，各级各部门还要不折不扣把国家和我省降低实体经济企业成本的各项政策落实到位。要积极化解企业融资难、融资贵难题，建立一季一次银企对接平台。市政府已设立了3个亿的转型发展基金，重点支持重大转型项目的发展。

　　各位代表，转型升级靠项目，没有项目等于零。我们要牢固树立服务项目就是服务转型的理念，要千军万马跑项目，要千辛万苦攻项目，要千方百计上项目。要用项目检验作风，用项目检验能力，以项目论英雄。只要我们咬定青山不放松，晋城就一定能够坚定地扛起转型升级、跨越崛起的重担，就一定能够坚实地走出转型升级、跨越发展的新路！

　　（二）开发区改革创新行动。

　　开发区的改革创新，是关系到开发区能否成为经济发展主战场的关键举措。改革创新的力度，决定着开发区发展的速度。要全力抓好国家级晋城经济技术开发区改革创新。按照省委省政府明确的“整合、改制、扩区、调规”的四大任务，必须聚焦招商引资、园区建设、服务企业“三大主业”，强力实施专业化、市场化、国际化的管理运行机制和实行领导班子任期制、全员岗位聘任制、绩效工资制的“三化三制”改革要求。4月份开发区“三化三制”改革要全部到位。要通过“管委会+公司”的市场化运作模式，整合北留周村煤电化工业园和巴公装备制造工业园区，年内要完成整合园区、“五规合一”和新区土地报批工作，形成新的“一区两园”的运行管理机制。各县（市）要积极申报省级开发区建设。结合全市产业发展规划，创新各县（市）园区管理体制和内部机制改革。坚持规划引领，3月底前完成制定细化落实措施，上半年完成县（市）开发区改革创新发展规划。推行“五规合一”，完善组织机构，创新突破“三化三制”要求，实行“开发区+开发公司”的模式，整合现有工业园区，形成错位发展、差异竞争的产业发展格局。高平、阳城今年完成申报省级开发区的报批任务。通过开发区的改革创新，我们努力把开发区全力打造成为转型升级的重要载体、区域经济的强力引擎和转型综改的主战场。

　　（三）招商选资引智行动。

　　招商选资引智是产业转型升级攻坚的关键之举，是我们解决经济结构性产业短板的重要举措。今年招商选资引智行动，要突出招新兴工业项目、高科技工业项目两大重点，这是今年招商选资的重中之重。要按照产业发展基础、产业发展特色和优势、需要什么项目、项目来时有什么支持政策和帮助举措四大目的去招商。要紧紧盯住高科技工业项目这个目标，围绕光机电、电子信息、高端装备制造、生物科技、节能环保、新能源、新材料等七大产业，进行系统化、定点式、有序性、精准对接的模式招商。要营造大招商招大商的浓厚氛围。坚持领导带头抓招商，抽调干部挂职招商、蹲点招商，在全社会营造重视招商、关心招商、人人招商的社会氛围。特别是要采取挂职招商方式，珍惜上海挂职招商的机遇，承接好“长三角”地区的好项目、大项目。各县（市、区）要发挥主观能动性，主动走出去、请进来，主动发挥各自积聚的人缘、地缘和乡缘，坚决盯住项目、盯住人才、盯住成果，真正实现招商选资引智结合起来；盯住长三角、珠三角、京津冀地区、中原城市群等重点区域，进行系统化、精准化招商，把招商选资引智作为增动能、补短板的重点工程，变成解决结构性矛盾和产业单一的突破口。要优化政策政务环境抓招商。加大对龙头企业、外来企业，特别是高科技企业的贴息、奖励和扶持。积极由一站式审批向代办式服务转化，由线下服务向线上服务转变。严格执行首问责任制、限时办结制、超时默认制等制度。凡是有利于项目建设的事，马上就办，涉及多个部门解决的事，要实行定时会商办，真正让项目投资者放心干事、安心创业。要扩大对外开放抓招商。采取以企招商方式，通过发挥广大在晋企业特别是世界500强、全国500强企业的人脉、信息、品牌等方面的独特优势，引进上下游配套企业，延伸产业链条，形成以企招商的“葡萄串”效应。积极主动召开晋城籍人士回家、创业、投资座谈会，热情邀请、广泛邀请吸纳国内外晋城籍的优秀企业家回乡创业。要创新制度抓招商。积极探索购买服务式的招商，对能够带动一个产业，特别是高科技产业的龙头企业，要实施一企一策，拿出更多的精力，投入更多的时间，用一切办法为项目落地扫清障碍、铺平道路。对中小企业，要在金融创新上下功夫，重点解决融资难、融资贵的问题。对成长型的小微企业，要以“双创”园区的建设和科技孵化基地的创新为依托，采取“创业苗圃-孵化器-加速器”阶梯式孵化体系，在新型创业高科技企业的孵化培育工作上下功夫。市“双创”协会，要发挥好郭台铭总裁、范守善院士和霍国庆教授担任顾问的契机，促进全市“双创”工作健康发展。确保让真正的创意企业能够迅速成长起来，从而加快促使创意变成产品、产品变为商品。

　　（四）城建质量提升行动。

　　城建质量提升是市域综合竞争力和城市综合承载力的发展要求，也是进入中原城市群发展规划核心发展区的重要门坎。要以人为中心，突出拓展、改造、提质，补齐补实城市发展短板。按照“十三五”时期建设“双百”规模大市区的目标，今年重点实施“双十”城市建设工程。加快城市道路建设，重点建设十大城市基础设施工程。新开工建设4街8路10互通，总长140.17公里。今年完成7街8路8互通，总长57.52公里。其中，富士康新区“四纵一横”道路连接工程，总长15.1公里，年内完成4项，重点完成黄华街南延、景西路南延、金鼎路南跨、金匠街西延；晋煤新区连接工程1项，道路总长5公里，为畅安路北延；主城区与东北部“三纵一横”连接工程，共4项，道路总长9.4公里，重点完成文博路北延、太岳街东延、畅安路南延、文化路全线贯通；主城区东南部街路微循环工程，道路总长4公里，重点完成山门街、塔东街、祥云街、太行南路建设。今年道路工程完工后，主城区将在现有“一纵一横”的基础上，新增文博路、景西路、太岳街“两纵一横”道路骨架工程，将形成新的“三纵两横”的主城区路网骨架格局。完善城市服务功能，重点实施十大公共基础设施建设。开工建设晋城大剧院、市民广场、市民公园，建成丹河水城音乐喷泉。实施晋城火车站、高速东口改造工程，完成高速南口提升改造。启动“大水源”工程，从高平引张峰水库优质水源进入市区，力争明年国庆前让市区群众喝上优质地表水。启动“大热源”工程，加快阳电向市区供热工程建设。启动“大气源”工程，开工建设阳城北留崇上至泽州南村门站高压长输管线，尽快实现双气源供气。十大城市公共基础设施工程，是关系市民生活质量、方便市民快进快出、改善市民候车条件，解决市民吃水、供热、用气的重大工程，我们一定要高标准、高质量完成，给市民提供一个舒心、便捷、干净、整洁、健康的生活家园。加强城市规划建设。要积极推行城市建设的“五规合一”总纲，6月底编制完成《美丽晋城战略规划》。要加快推进市区城中村改造步伐。新开工建设棚户区改造801套，年内建成1500套。提升城市管理水平。深入推进城市执法体制改革和文明城市创建工作，集中开展市容环境大整治。加大城市交通管理执法力度，优化重点区域、重点时段交通秩序。落实公交优先战略和公交畅通工程，增加公交夜班延长营运时间线路，年内新增新能源公交车100台。积极推进城市地下综合管廊和海绵城市建设，积极创建国家节水型城市。努力打造特色城镇。要持续加快大县城和重点城镇建设。各县（市）要突出功能分区，重点完善市政配套和服务功能，加大边街小巷、环境卫生、交通秩序等整治力度，进一步改善人居环境。扎实推进重点城镇建设，围绕全域旅游，打造一批商贸物流、休闲旅游、山水田园、历史文化等特色小镇，继续抓好巴公国家新型城镇化综合试点工作。通过城建质量提升行动，努力把中心城区和县城，建成交通快捷、功能完善、环境舒适、品位提升、生态优美的宜居宜业宜游城市，实现城市面貌一年一个样、五年大变样！

　　（五）通道快捷建设行动。

　　通道快捷建设是连接城市内外的重要纽带，也是人流、物流的重要载体，也是加快解决“进不来、出不去”的交通瓶颈的主要举措。提高城市交通承载能力，让城市更加“舒经活络”，是通道快捷建设的主要内容。畅通外联交通“大动脉”。按照“十三五”提出的建设“双百”大市区的目标，加快打造“铁、公、机”立体综合交通体系，实现交通提质提效。认真做好民用机场建设前期工作，年底前争取列入《2016-2025年全国民用机场布局规划》。加快推进太焦高铁建设，认真做好郑银高铁在我市境内的前期准备工作。继续推进阳蟒高速项目建设，积极协调高陵高速河南段建设，高沁高速年底前正式通车。晋阳一级路东沟至周村段年内建成通车。构建内通“三环”大格局。确保3年完成“三环”连通工程。开工建设晋阳晋济连接线，与环城高速相连接，确保今年建成便捷通畅的高速大外环（三环）。开工建设36公里的陵沁路西北环改线项目，加快推进207国道改线及8个互通工程，确保2018年建成城市新外环（二环）。启动现状陵沁路西北环城市道路化改造，实施白水街和金凤路延伸与改造，确保2019年建成城市新内环（一环）。优化互联互通“毛细管”。加快推进国道207线新房洼至省界瓶颈路段改造工程。启动长晋二级路、省道坪曲线改造工程，认真做好丹河快线工程项目前期工作。围绕全域旅游大通道建设，加快完善通达县城、中心镇及重要旅游景区的公路连接线。推进市域县乡公路和农村公路建设。今年，要通过推进通道大建设，实施交通大会战，全面优化路网结构，加快打造外部配套成环、内部纵横成网的大进大出、快进快出的大格局，扎实奠定“双百”规模大市区建设的框架。

　　（六）全域旅游打造行动。

　　全域旅游打造行动是带动旅游业发展成为支柱产业的重大龙头。今年，我们要围绕“十三五”时期创建国家全域旅游示范区的目标，以建设太行屋脊全域旅游大通道、天下公园生态观光大走廊、举办首届全域旅游嘉年华为主要抓手，实施2017年全域旅游打造行动。一是启动建设总长约500公里，途经陵川、泽州、阳城、沁水、高平5县（市），覆盖30个乡镇、262个行政村、26万人口，串联7个旅游片区23个旅游景点的太行屋脊全域旅游大通道，10月份完成项目前期、开工建设。通道将辐射太行古八陉中的3条古陉、10条古道、17个关隘和14个全国重点文物保护单位、6个省级保护单位，3个中国历史文化名镇、7个中国历史文化名村、2个省级历史文化名镇、10个省级历史文化名村，18个古八景，46个自然名胜和风景点。这条500公里的大通道将连通晋城的山山水水，连通晋城的历史文化、人文荟萃。这条大通道将是承载晋城五千年灿烂文明的时光隧道，也是建成小康社会、实现“中国梦”的振兴大道、康庄大道。我们要高标准把大通道建成晋城一条亮丽的风光大道，让大通道展现“风光绣进城市，通道传承历史，城市留住记忆，太行再现辉煌”的全域旅游文化灵魂的支柱引领作用。二是开工建设总长37公里，途经吴王山、玉屏山、白马寺、司徒小镇、龙门、丹河湿地的天下公园生态观光大走廊，将主城区周边分散的景区、景点、公园通过观光走廊的形式串联起来，搭建起一条连续不间断“景观廊道观光带”。三是举办晋城首届全域旅游嘉年华，把全域旅游嘉年华打造成一场彰显晋城魅力的全域旅游盛会。

　　同时，通过实施“大通道、大走廊、嘉年华”，带动全市旅游体制机制、顶层设计、基础设施、景点景区、宣传营销各个方面全面提升。今年，要成立市旅游发展委员会，2月底前各县（市、区）旅游发展委员会列入政府组成部门，并完成机构、人员、职能整合。列入《全省旅游景区（景点）体制机制改革创新工作推进方案》的16家旅游景区，在6月底前完成景区体制机制改革任务。2月底要编制完成《沁河干流古村落文物密集区保护利用总体方案》，争取国家立项。5月底编制完成《晋城市全域旅游发展五年规划》。新建、改建高标准旅游厕所60座，每个县（市、区）完成10座2A级以上旅游厕所。开通4A级以上景区的直通车或常态化的客运公交专线，完成县道以上公路旅游标识标牌全覆盖，构建省内一流的全域旅游导示系统。完成北留、大阳、神农3个特色旅游乡镇，司徒、东四义、冶底村等30个特色旅游村，以及皇城相府、大阳古镇、司徒小镇等300个特色乡村旅游客栈建设。依托中原经济区和京津冀地区，多管齐下，构建立体式、辐射型宣传促销模式。全力打造“全域布局、全景覆盖、全局联动、全业融合、全民参与”的大旅游格局，把全域旅游培育壮大为我市的优势主导产业。

　　（七）精准脱贫攻坚行动。

　　以脱贫攻坚为重点，以深入推进农业供给侧改革为主线，将脱贫攻坚与“三农”工作紧密结合起来，促进农业增效、农民增收。深入推进农业供给侧改革。依托我市特色现代农业发展的良好基础，明确县区功能定位，优化产业发展布局，推进优势产业集聚。深入推进农产品加工“513”工程，培育亿元以上龙头企业14家。实施“品牌”战略，新增1-3个著名商标或名牌产品。推进美丽乡村建设，以城郊城镇化带动型、工矿企业支撑型、现代农业产业发展型、农旅一体乡村旅游型等模式，创建市级美丽宜居示范村20个。加快培塑家庭农场、规模养殖场、专业大户、农民专业合作社、农业园区等新型农业经营主体，完成新型职业农民培训3000人。建立和完善市、县两级农产品质量安全监管与市场信息、农业执法、安全认证、检验检测“四位一体”的农产品质量安全监管体系，农产品抽检合格率稳定在96%以上。实施八大产业扶贫工程。实施畜牧产业扶贫工程，在贫困村新发展养羊存栏2万只、养牛存栏500头、出栏生猪5万头、养蜂5000箱；实施蔬菜产业扶贫工程，新发展设施蔬菜（食用菌）2000亩；实施干鲜果产业扶贫工程，提质增效干水果5万亩；实施蚕桑产业扶贫工程，新发展桑园3000亩；实施小杂粮产业扶贫工程，涉及贫困户人均1亩种植面积；实施中药材产业扶贫工程，新发展中药材3万亩；实施休闲农业和乡村旅游工程，在贫困县创建200个省级休闲农业与乡村旅游示范点、200个高标准“农家乐”；实施农产品电商扶贫工程，在每县建设1个电子商务服务中心，乡村站点超过1000个。扎实推进易地搬迁扶贫。坚持易地搬迁扶贫与新型城镇化建设、产业发展、旧村开发和村庄撤并、社会保障相结合，妥善解决搬迁群众的居住、看病、上学等问题，统筹谋划安置区产业发展与群众就业创业，完成建档立卡贫困人口易地扶贫搬迁6511人，同步搬迁916人，确保搬迁对象搬得出、稳得住、能致富。推进贫困地区基础设施建设。扎实推进生态补偿扶贫，通过支持贫困人口转化为护林员等措施，带动全市684人稳定脱贫。完成农村公路建设72公里，完成沁水、陵川13个乡镇、26个贫困村的26处饮水安全配套改造工程，巩固提升1.03万人的饮水安全保障水平。确保今年完成84个村、2.54万人的脱贫任务，沁水、陵川两个贫困县脱贫摘帽，其它非贫困县全部完成贫困村、贫困人口脱贫。

　　（八）高端人才引进行动。

　　高端人才引进行动是解决我们人才资源稀缺最重要的举措。在高端人才引进方面，要重点实施三大工程，真正实现人才与发展比翼齐飞。实施引智政策配套工程。以“服务于一流人才”为目的，3月份开工建设“梧桐苑”人才房项目，其中建设60套高端人才引进房工程，进一步让一流人才留得住。研究制定一流人才引进政策，为来晋城工作和创业的一流人才队伍解决后顾之忧。对于特别优秀的急需人才，实行“一人一事，一事一策”，用真心真情服务人才、留住人才。实施项目科技孵化工程。支持引导社会资本建设“众创空间”、“星创天地”等创新创业平台，丰富一流人才创新创业载体。晋城市科技研发基地（科技企业孵化器）项目9月份竣工，积极为入驻企业提供物理空间和完备的科研基础设施，降低创业者的创业风险和创业成本。按照“专业服务+天使基金+投资导师”模式，为科技企业提供资金支持、引导支持，让有潜力的企业在孵化器内成功“孵化”，发展壮大。今年，将重点引进清华碳纳米研发项目。实施成果转化工程。加快百名院士博士工作站的建设，引导高校及其科研人员参与到晋城创新创业，不断解放和激发科技潜能，推动提高科技成果转化率。今年争取富士康纳米光机电高科技研发中心、国际煤层气产业联盟、国家级环境保护环境感官应激与健康重点实验室三大院士工作站落户晋城。要通过人才引进行动，吸引高端人才来晋城创业，把晋城打造成山西乃至全国最具硅谷气质的“人才之城”、“爱才之城”，让“近者悦、远者来”，让更多的专家和人才向往晋城、爱上晋城、扎根晋城。

　　（九）生态环境治理行动。

　　生态环境是一个地方的核心竞争力。生态环境是晋城的最大优势、最大财富、最大品牌。要牢固树立绿水青山就是金山银山的理念，坚持保护和治理并重，推动生态环境建设再上新台阶。强力推进大气污染防治。扎实开展“铁腕治污”专项治理，深化“减煤、降尘、控油、治烟”重要举措，持续削减污染物排放量，对市区内未实施集中供气的25531户居民、未实施集中供热的4295户居民实施集中改造。全面取缔市区20蒸吨及以下燃煤锅炉8台、县城建成区10蒸吨及以下燃煤锅炉和茶浴炉80台，完成老旧车淘汰任务。强力推进水污染防治。实施市区、高平、阳城生活污水处理厂扩容工程，加快丹河高平河西段污染治理步伐，完成城区西河、书院河等10条黑臭水体治理。加大环境执法监管力度，坚决打击各类环境违法行为。强力推进生态文明建设。继续实施林业“六大工程”，完成营造林工程1.17万亩，村庄绿化工程40个，环城生态圈新增绿化面积1.3万亩。积极推进沁（丹）河流域生态修复与保护工程建设，完成水土流失治理12万亩。强力推进低碳循环发展。加快推进国家低碳城市试点工作，扎实开展国家循环经济示范城市创建。大力支持清洁能源普及，积极推广新能源汽车，年底要实现全市电动汽车充电桩全覆盖。

　　各位代表，珍视生态，就是珍视我们的生命；建设生态，就是建设我们的家园。我们要通过生态环境治理行动，把晋城的生态优势转化为竞争优势，着力打造山清水秀、天蓝地绿的美丽家园！

　　（十）民生福祉改善行动。

　　改善民生是我们工作的宗旨，也是最大的政绩。今年在民生福祉改善行动中要抓好五个方面的工作，让老百姓享受真真切切的实惠，得到实实在在的好处。教育方面，在市区新建现代化双语幼儿园1所，全市新建改建幼儿园12所。陵川县要通过国家义务教育发展基本均衡督导认定。启动高中办学条件标准化行动。成立制造业教育集团。开工建设太原科大晋城校区图书馆、实训中心、科研实验室建设，扩展学科专业，更好服务于地方经济。医疗卫生方面，以高平县乡医疗卫生机构一体化改革为抓手，推动全市医改一体化工作全面完成，提升基层医疗服务能力。开工市人民医院扩建工程和市中医院建设工程。文化方面，加大史志资源开发利用力度，挖掘文化宝藏，启动优秀传统文化传承工程和乡村文化记忆工程，重点做好“太行屋脊大通道”沿线景区、村镇的文化资源挖掘整理和保护传承。持续实施文化惠民工程，继续完善市、县、乡、村四级基础公共文化活动场所建设，大力扶持文化精品创作，广泛开展群众性文体活动，进一步满足群众日益增长的精神文化需求。社会保障方面，继续提高城乡低保标准，特困人员供养标准每人每年提高500元。整合新农合和城镇居民医疗保险两项制度，建立全市统一的城乡居民基本医疗保险制度，实现覆盖范围、筹资政策、保障待遇、医保目录、定点管理、基金管理“六统一”和城乡居民一体化管理服务，使城乡居民得到更多实惠。继续推进城乡社区老年日间照料中心建设，探索推动公建民营、股份制、PPP模式等养老服务新模式，引导和鼓励社会力量积极兴办养老福利事业。举办全市创业产品展和创业大赛，利用晋城人才网的服务功能，同步举办线上线下各类招聘会，全市城镇新增就业达到3.4万人，转移农村劳动力2.3万人，城镇登记失业率控制在4.2%以内。社会管理方面，深化“平安晋城”创建活动，强化源头治理，严格落实社会稳定风险评估制度，坚决防止大规模群体性事件的发生。严厉依法打击各类犯罪活动，深入开展打黑除恶专项斗争，集中打击电信网络诈骗犯罪，进一步加强社会治理防控体系建设，持续推进视频监控“天网工程”建设，完善并推广“雪亮工程”经验做法。扎实开展安全生产月、安全生产咨询日活动，提升全民安全意识和安全素质。以煤矿、非煤矿山、危险化学品、烟花爆竹、道路交通、冶金工贸行业、职业健康防护为重点，进行全方位的安全隐患排查治理，坚决做到减少一般事故，遏制较大事故，杜绝重特大事故，为全市经济社会发展创造安全、稳定的社会环境。

　　此外，还要发展妇女儿童、老龄、慈善和红十字会等事业。加强气象、地震、人防、档案、民族宗教、外事、侨务等工作。加强国防动员和双拥工作，积极支持驻市部队改革，促进军民融合深度发展。

　　在做好以上各项工作的同时，市政府还要为民办好九件实事：一是实施残疾预防重点干预和残疾儿童抢救性康复项目；二是为全市城乡怀孕妇女提供免费产前筛查和诊断服务；三是新建农村老年人日间照料中心；四是免费送戏下乡；五是农村义务教育寄宿制学生营养改善全覆盖；六是贫困地区农村饮水安全提升全覆盖；七是实施遗传性耳聋基因免费筛查项目；八是实施农村贫困户危房改造保障工程；九是全市建档立卡贫困人口和农村低保人口参加大病医疗商业补充保险。

　　这九件实事，件件都连着广大人民群众的切身利益。我们一定要本着以人民为中心的理念，急群众之所急，想群众之所想，把实事办好、好事办实，切身提升人民群众的幸福感，让更多的改革成果惠及到广大人民群众，切身增强人民群众的获得感。

　　三、加强政府自身建设

　　2017年是抓落实、抓攻坚之年，是全面提高工作标准、全面提高工作成效之年。面对新的形势和任务，各级政府必须加强自身建设，切实提高谋划力、担当力、落实力、约束力，为全市转型升级攻坚提供有力保障。

　　提高谋划力。干在实处永无止境，走在前列敢谋新篇。要按照“大开放、大转型、大创新”发展思路，善于站在全局和战略的高度谋划发展，把工作标准与中央要求“对表”、与发达地区对标、与国际通行惯例对接，立足当前、着眼长远，切实提高工作标准，做到立意高、落点实、操作性强。要切实克服思想懈怠、自我满足、自我放松的倾向，把工作标准定在干就干到最好上，定在积极作为、奋力赶超上。先行而不浮躁，争胜而不死板，一定要实现政府各项工作样样争一流，勇当排头兵。

　　提高担当力。今年是全市转型升级发展的攻坚之年。要大力倡导“闯”的精神，聚焦聚力聚神抓好事关全市发展大局的改革事项，在产业转型、国企改革、招商引资等方面要解放思想，提高认识，既要摆出不服输、扛得住的身段，更要有多吃螃蟹、能啃硬骨头的手段。要有强烈的“我要干”的意识，不等不靠，拿出夜以继日、夙夜在公的精气神，以时不我待，只争朝夕的劲头，担当作为。有困难，上；有责任，扛；有软肋，强。撸起袖子、挽起裤腿、咬紧牙关加油干。在赛场上跑，在跑场上见，确保跑到位，当冠军。要真诚做事、诚信服务，严格履行向社会作出的承诺。凡确定和承诺的事、安排部署的工作，必须将落实进行到底，以转型升级发展的实际成效推动全年经济社会发展。

　　提高落实力。一分部署，九分落实。一打纲领不如一个行动。说的再好，没有落实等于零。要坚决按照习总书记要求，亲力亲为抓改革，扑下身子抓落实。各项工作都要按照“建立台账，跑表计时，到点验收”的机制抓好落实。要严格落实省政府“13710”工作制度，今年6月底前市县乡三级政府全面建成电子督办平台，将重点工程项目、企业技改项目和招商引资项目纳入平台管理，推动政府各项工作见人见事、见根见果，抓细抓小、抓紧抓实。发挥政府督查作用，明确督查的工作重点，盯住关键环节，查安排、查进度、查成效，确保各项工作任务全面及时高效落实。同时，要进一步加大追责问责力度，做到政府工作有清单、有督查、有考核、有问责，确保中央、省、市各项决策部署落实到位。

　　提高约束力。坚决落实全面从严治党的各项要求，全面履行管党治党的主体责任，在市政府组成部门设立党组（党委）办，切实抓好“三基”工作，全面加强政府系统党的建设。要按照干部清正、政府清廉、政治清明的要求，落实“两个责任”，坚持“一岗双责”，深入推进政府系统党风廉政建设和反腐败斗争。全面推进政务公开，实现决策、执行、结果公开透明。强化财政支出、工程建设、公共资源交易等重点领域和重要环节的监管，切实扎牢制度笼子。牢固树立“过紧日子”思想，完善厉行节约长效机制，严控“三公”经费支出，以清正廉洁的良好形象促进发展、取信于民。

　　各位代表，光荣的使命鼓舞我们砥砺奋进，人民的期盼鞭策我们勇往直前。让我们更加紧密地团结在以习近平同志为核心的党中央周围，在省委、省政府和市委的坚强领导下，坚定信心、主动作为，只争朝夕、狠抓落实，全面完成各项目标任务，以实际行动和出色业绩迎接党的十九大胜利召开!
